# Dangerous passage: the utility and accuracy of modern chest computed tomography in penetrating thoracic injuries with potential transmediastinal trajectory

**DOI:** 10.1007/s00068-023-02315-8

**Published:** 2023-06-26

**Authors:** Marco Sozzi, Kenji Inaba, Morgan A. Schellenberg, Kazuhide Matsushima, Matthew J. Martin

**Affiliations:** Los Angeles County + USC Medical Center, 2051 Marengo Street, Room C5L100, Los Angeles, CA 90033 USA

**Keywords:** Penetrating trauma, Chest trauma, Transmediastinal gunshot wounds, Computed tomography

## Abstract

**Aim:**

The aim of this study is to evaluate utility and reliability of chest CT as a standalone screening modality for stable patients with thoracic GSWs and potential transmediastinal trajectories.

**Methods:**

All patients with thoracic GSWs over a 5-year period were identified. Unstable patients requiring immediate surgery were excluded and the remaining underwent chest CT with intravenous contrast. Sensitivity and specificity for clinically significant injuries were tested against an aggregate gold standard of discharge diagnosis including imaging, operative and clinical findings.

**Results:**

A total of 216 patients met inclusion criteria and underwent chest CT. After imaging, 65 (30.1%) had indication for immediate surgery, of which 10 (4.6%) underwent a thoracic procedure for chest injuries while 151 (69.9%) were selected for nonoperative management (NOM). 11 (5.1%) required a delayed thoracic operation, none due to injuries missed on CT. The remaining 140 (64.8%) underwent successful NOM. Up to 195 (90.3%) patients had successful NOM of thoracic injuries. Only 9.2% required additional imaging, all negative. CT identified a cardiac injury in one case and a vascular injury in two cases, all confirmed by surgery, while one thoracic IVC injury missed on CT was found intraoperatively. 2 patients had CT suspicious for esophageal injury, ruled out by following investigations. There was one death in the total cohort, none in the NOM group.

**Conclusions:**

Modern high-quality CT provides highly accurate and reliable screening modality for penetrating chest and mediastinal injuries and can be used as a standalone study in most patients or to guide further tests. Chest CT facilitated successful NOM.

## Introduction

Ballistic injuries to the chest may traverse and damage mediastinal structures, with reported mortality rates of 33–79% among the patients that reach the hospital alive [1–5]. Although in many cases of confirmed transmediastinal trajectory the patient will present with hemodynamically instability or as a transient responder, there is evidence that up to 80–85% of patients with a thoracic gunshot wound (GSW) [6] and a proportion ranging between 12 and 51% with mediastinal involvement present in a stable condition [1–5]. These patients can benefit from an appropriate workup to guide the management, that classically involved multiple studies, including echocardiography, upper endoscopy and/or esophagography, catheter-based angiography, and computed tomography (CT) [1, 5, 7].

Previous studies have evaluated the role of CT scan in the detection of bullet proximity to the mediastinum, indicating the need for additional investigations [5, 8–11], but no large, the recent series have assessed utility and reliability of chest CT scan and CT angiography as a standalone initial screening and imaging modality. Our hypothesis is that modern chest CT can be safely and accurately utilized to exclude significant injuries and identify patients that need a surgical intervention, guiding further workup in a selected proportion of patients.

## Methods

All patients with a thoracic or thoracoabdominal gunshot wound (GSW) presenting to our Level I trauma center between January 2017 and December 2021 were included in this retrospective study. The trauma registry was queried to identify all subjects and then a detailed medical record and radiologic study reviews were performed to confirm chest involvement. The chest was defined anatomically as inferior to the clavicles anteriorly and to the superior aspect of the scapula posteriorly, and superior to the costal margin inferiorly. All gunshot injuries including those to extra-thoracic locations, along with all retained fragments were documented. Patients in which chest involvement was not confirmed by review of medical charts, operative records and imaging reports were excluded.

The clinical management, including diagnostic workup, and the results of all imaging were collected from the detailed chart and imaging review. Patients were stratified according to their immediate management. Those with hemodynamic instability that required a resuscitative thoracotomy in the trauma bay or were immediately brought to the operating room for emergent surgery were excluded from further analysis. Patients declared dead on arrival and asymptomatic patients with superficial injuries, who were discharged without imaging or intervention were also excluded. All other patients underwent primary and secondary evaluation in the trauma bay, including plain X-rays and extended focused assessment with sonography for trauma (eFAST), followed by a chest CT scan with intravenous contrast. The final study population included all patients with a GSW to the chest, evaluated with a chest CT scan. CT evaluation included multiplanar scans of chest and other body areas, as suggested by mechanism of injury or clinical evaluation. If suspicion for arterial injuries was high, timing for contrast injection was set up to perform a CT angiography. When demonstration of bullet trajectory with intravenous contrast scans was not sufficient to rule out esophageal involvement, additional scans with oral contrast swallow were performed if patients’ clinical condition was felt to be stable. Performance metrics of chest CT scan for the detection of clinically significant injuries (sensitivity, specificity, accuracy, positive predictive value (PPV) and negative predictive value (NPV)) were calculated and were tested against an aggregate gold standard of final discharge diagnosis including all imaging and operative findings.

The primary outcome of the study was accuracy and reliability of the initial chest CT scan as a diagnostic study and for determining disposition. Secondary outcomes were the performance characteristics of any secondary diagnostic tests other than CT scan that were performed to evaluate thoracic and mediastinal structures. Descriptive statistics were performed using IBM SPSS Statistics for Windows (Version 28, IBM Corp.). Continuous variables are presented as median and interquartile range. Categorical variables are presented as number of occurrences with percentage of their group. Univariate comparisons were performed with Student’s *t* test, chi-square, or Mann−Whitney *U* test as appropriate. Appropriate Institutional Review Board (IRB) approval was obtained for this study (HS-22-00241).

## Results

**Fig. 1:**
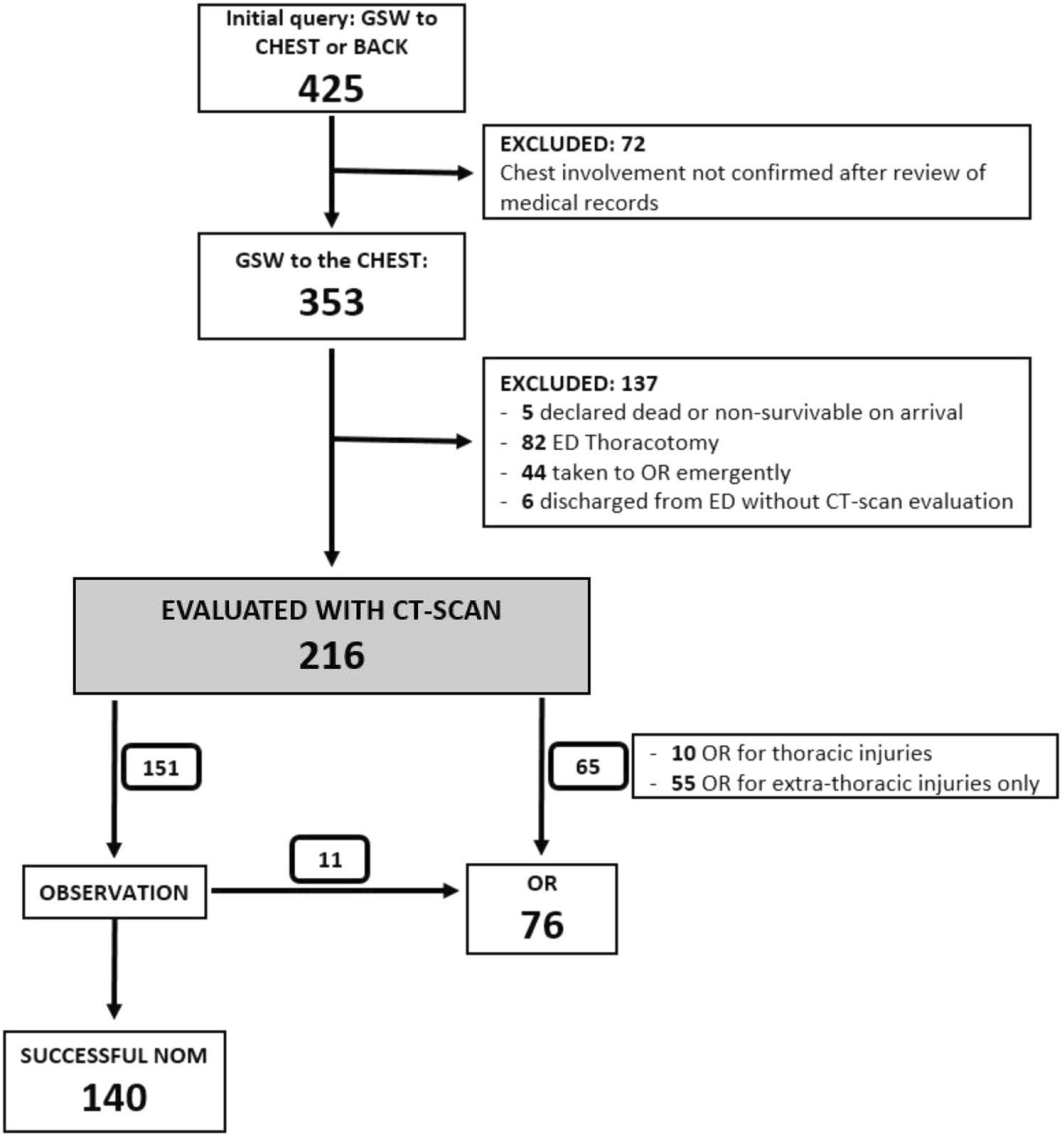
The study flowchart showing included and excluded patients. *GSW* gunshot wound, *ED* emergency department, *OR* operating room, *NOM* nonoperative management

Overall, 425 patients were admitted with a GSW to the chest or back. Review of medical records confirmed chest involvement in 353 patients. A total of 137 patients did not undergo a CT-scan evaluation due to hemodynamic instability requiring immediate surgery, because declared dead or nonsurvivable on arrival or because CT scan was deemed unnecessary before discharge and were excluded. The remaining 216 patients that underwent a CT scan were included in the final study population. The study flowchart in Fig. 1 shows details of the exclusion process and management of included patients. Demographic characteristics, clinical conditions on arrival and injury severity are shown in Table 1. The majority of patients were male (90%), had a median age of 28 years, and arrived via EMS transport (74%). Almost all (95%) were triaged as full trauma team activations, 8.3% were hypotensive on arrival, and the median injury severity score (ISS) was 10. As per the study inclusion criteria, all patients were deemed stable for further imaging after the initial evaluation and resuscitation, and underwent CT scan of the chest with intravenous contrast as well as simultaneous CT scan of any other anatomical area of urgent interest.

**Table 1:** Demographic characteristics, clinical conditions and injury severity

	*N* = 216
Male, *n* (%)	194 (89.8)
Age, years, median [IQR]	28 [15]
Ethnicity, *n* (%)	
Asian/Non-Pacific Islander	4 (1.8)
Black/African American	20 (9.3)
Hispanic/Latino	157 (72.7)
White	21 (9.7)
Other	14 (6.5)
Entry mode, *n* (%)	
EMS	160 (74.1)
Non-EMS	25 (11.6)
Transfer	31 (14.3)
TTA, *n* (%)	206 (95.4)
SBP < 90, *n* (%)	18 (8.3)
GCS < 9, *n* (%)	5 (2.3)
ISS, median [IQR]	10 [14]
ISS, *n* (%)	
≤ 14	145 (67.1)
15–24	41 (19.0)
≥ 25	30 (13.9)
Max AIS head and neck ≥ 3, *n* (%)	1 (0.4)
Max AIS face ≥ 3, *n* (%)	1 (0.4)
Max AIS chest ≥ 3, *n* (%)	115 (53.2)
Max AIS abdomen and pelvis ≥ 3, *n* (%)	40 (18.5)
Max AIS extremity ≥ 3, *n* (%)	23 (10.6)
Max AIS external ≥ 3, *n* (%)	2 (0.9)

*IQR* interquartile range; *EMS* emergency medical services; *TTA* trauma team activation; *SBP* systolic blood pressure; *GCS* Glasgow Coma Scale; *ISS* Injury Severity Score; *AIS* Abbreviated Injury Scale

After CT scan, 65 (30.1%) patients had indication for immediate surgery. Of these, 55 (25.4%) were transferred to the OR exclusively due to injuries involving abdomen, neck or extremities and did not undergo any surgical procedure involving the chest, that was managed nonoperatively. The other 10 (4.6%) patients received indications to operative management of thoracic injuries, including large hemothorax with active contrast extravasation on CT scan in 7 patients, pericardial bleeding, aortic arch pseudoaneurysm and subclavian artery pseudoaneurysm with arteriovenous fistula. Table 2 shows the details of management and procedures performed.

**Table 2:** Management and procedures

	*N* = 216
OR after CT scan, *n* (%)	65 (30.1)
Thoracic procedure	10 (4.6)
Extra-thoracic procedure only (NOM of chest injuries)	55 (25.4)
Time to OR for immediate surgery, minutes, median [IQR]	140 [924]
NOM attempt, *n* (%)	151 (69.9)
Successful NOM, *n* (%)	140 (64.8)
NOM attempt of thoracic injuries, *n* (%)	206 (95.4)
Successful NOM of thoracic injuries, *n* (%)	195 (90.3)
Delayed thoracic surgery, *n* (%)	11 (5.1)
Time to OR for delayed surgery, minutes, median [IQR]	4400 [8184]
Procedures, *n* (%)	
Left thoracotomy	3 (1.4)
Right thoracotomy	8 (3.7)
Sternotomy	2 (0.9)
Laparotomy	26 (12.0)
Laparoscopy	5 (2.3)
Thoracoscopy	7 (3.2)
Neck exploration	2 (0.9)
Extremity exploration	25 (11.6)
Other	9 (4.1)
Endovascular surgery	1 (0.4)
IR procedure	16 (7.4)

*OR* operating room; *NOM* nonoperative management; *IR* interventional radiology

The remaining 151 (69.9%) patients were selected for nonoperative management (NOM). Among these, 11 (5.1%) required a delayed thoracic operation, while the other 140 patients were successfully managed nonoperatively. Indications for delayed surgery are shown in Table 3. When considering the management of chest injuries alone regardless other body areas, up to 195 (90.3) patients were successfully managed nonoperatively. The GSW involved the mediastinum in 13 (6.0%) patients, as proven by CT scan evaluation of bullet trajectory. Heart ultrasound was performed in 28 (12.9%) patients, while 20 (9.2%) patients required additional imaging, including esophageal studies and bronchoscopy, all of which were negative for significant thoracic or mediastinal injuries.

**Table 3:** Indication for surgery and procedures performed in 11 patients that underwent a delayed thoracic operation

Retained hemothorax/MINI-THORACOTOMY(1) and VATS(4)	5
Hemothorax with high output chest tube/THORACOTOMY	2
Intrapericardial foreign body removal/VATS and VATS + THORACOTOMY	2
Bronchopleural fistula and persistent PNX / VATS	1
Bilothorax, concern of diaphragmatic injury (positive) / VATS + LAPAROSCOPY	1

*VATS* video-assisted thoracic surgery; *PNX* pneumothorax

The final diagnoses of injured organs and workup information are listed in Table 4. In three cases chest CT scan successfully identified a cardiac or vascular injury that was later confirmed by surgical exploration. These included a patient with evidence of pneumomediastinum, pneumopericardium and active pericardial contrast extravasation, who underwent a sternotomy to control the pericardial bleeding, finding a contusion of the right ventricle extending towards the inferior vena cava (IVC), with no defect in cardiac wall. The second patient had a pseudoaneurysm of the aortic arch and underwent an endovascular repair (TEVAR). Finally, in one patient CT scan showed a pseudoaneurysm of subclavian artery with arteriovenous fistulization to subclavian vein. This underwent a sternotomy with ligation of subclavian vein and repair of subclavian artery with reverse saphenous vein graft interposition.

**Table 4:** Final diagnosis, imaging studies and consequences of abnormal exams

	*N* = 216
Chest cavity penetration, *n* (%)	127 (58.8)
Mediastinum involvement, *n* (%)	13 (6.0)
Injuried organs and structures, *n* (%)	
Heart	0 (0)
Thoracic aorta	1 (0.4)
Pulmonary vessels	0 (0)
SVC	0 (0)
Thoracic IVC	1 (0.4)
Other thoracic vessels (subclavian artery, internal mammary artery)	2 (0.9)
Esophagus	0 (0)
Trachea and bronchi	0 (0)
Lungs	104 (48.1)
Diaphragm	27 (12.5)
Spine	32 (14.8)
Spinal cord	17 (7.8)
Heart US, *n* (%)	28 (12.9)
Abnormal	2 (0.9)
CT scan, *n* (%)	216 (100)
Abnormal	137 (63.4)
Esophagogram, *n* (%)	8 (3.7)
Abnormal	0 (0)
Esophageal contrast CT, *n* (%)	5 (2.3)
Abnormal	0 (0)
Esophagoscopy, *n* (%)	4 (1.8)
Abnormal	0 (0)
Bronchoscopy, *n* (%)	3 (1.4)
Abnormal	0 (0)

*SVC* superior vena cava; *IVC* inferior vena cava; *US* ultrasound; *CT* computed tomography

There was one significant vascular injury that was coded as a missed injury by the initial chest CT scan. This was a contained thoracic inferior vena cava (IVC) injury without active hemorrhage that was missed on the preoperative CT scan and identified intraoperatively. This patient initially underwent an exploratory laparotomy to address multiple intrabdominal injuries, and then later developed a massive hemothorax requiring a thoracotomy, with identification of an IVC laceration that was repaired primarily. Two patients had chest CT scans suspicious for a possible esophageal injury, but were ruled out intraoperatively in one patient and by endoscopy in the other. No CT scan raised a concern for tracheobronchial injuries and there were no subsequent tracheal or bronchial injuries identified. No injuries to the esophagus or tracheobronchial tree were diagnosed later during hospital course in patients with a negative initial chest CT scan.

Performance metrics of CT scan were calculated testing its findings against the final diagnoses obtained by clinical and operative evidence and by all other imaging studies. Results are shown in Table 5 and are additionally broken down by anatomic mediastinal structures of interest. The overall performance demonstrated a sensitivity of 75%, specificity of 99.1%, accuracy of 98.6%, PPV of 60%, and NPV of 99.5%. The accuracy for individual mediastinal structures ranged from 99.1 to 100% and the NPV from 99.5 to 100%. In this series, there was one death (0.4%) in the total cohort, while no patient died in the NOM group. The one death was a patient with a massive hemothorax and concomitant spinal cord injury who underwent immediate thoracotomy and lung resection. Postoperatively the patient then developed severe pneumonia with hypoxic respiratory failure and eventual cardiac arrest several days after surgery. Among the 37 (17.1%) patients requiring mechanical ventilation, median ventilator days were 2 (IQR = 3), while overall need for intensive care unit (ICU) admission was 119 (55.1%), with a median ICU length of stay of 4 days (IQR = 4). Overall median hospital length of stay for the study population was 5 days (IQR = 9).

**Table 5:** Performance metrics of chest CT scan for clinically significant injuries

	TP	TN	FP	FN	Sensitivity	Specificity (%)	Accuracy (%)	PPV	NPV (%)
HEART	1	215	0	0	100%	100	100	100%	100
VESSELS	2	213	0	1	66.7%	100	99.5	100%	99.5
ESOPHAGUS	0	214	2	0	–	99.1	99.1	0	100
TRACHEA AND BRONCHI	0	216	0	0	–	100	100	0	100
OVERALL	3	210	2	1	75%	99.1	98.6	60%	99.5

*TP* true positives; *TN* true negatives; *FP* false positives; *FN* false negatives; *PPV* positive predictive value; *NPV* negative predictive value

## Discussion

Proper evaluation and management of the small proportion of patients presenting with a thoracic GSW and not requiring emergency surgery is still largely unreported. The existing evidence about diagnostic imaging studies to rule out potential mediastinal injuries is mainly based on the small series that are over a decade old and utilized earlier generation CT scan platforms, frequently involving multiple modalities and invasive methods such as angiography and aortography, endoscopic evaluation of esophagus and tracheobronchial tree or swallow studies. The debate about prioritization of evaluation of vascular structures or aero-digestive tract [1, 2] has been largely overcome in many modern trauma centers with the advent of high-quality CT-scan with intravenous contrast, that allows the simultaneous study of the whole chest, providing for more selective use of subsequent directed diagnostic procedures or imaging modalities.

In 1998, Grossman [8] presented a series of 15 stable patients with GSWs involving the chest, showing that patients in which CT scan excluded mediastinal trajectories were safely managed without additional invasive diagnostic studies. Burack et al.[4] described their practice in the management of patients with a penetrating mediastinal injury, including 135 stable patients evaluated with a CT angiography (CTA), that underwent a formal angiography only in case of abnormal mediastinal findings on CTA. Similarly, other retrospective series evaluating stable patients with transmediastinal GSWs [9, 10] found that CT scan safely allowed to prevent further diagnostic studies when conclusively negative, while patients with suspicious findings underwent other imaging studies. Interestingly, in these series the CT scan was never considered conclusively diagnostic in presence of abnormal findings, and patients always underwent further tests before determining the presence of an injury requiring surgical treatment. In 2000, Hanpeter et al.[11] presented their prospective analysis of the role of CT scan in the evaluation of 24 stable GSW patients with a clinical suspect of mediastinal involvement. In 22 patients the missile trajectory was accurately determined by imaging. Moreover, the CT scan was deemed able to determine a significant change in management in half of these patients, including one who was transferred to the operating room based on the CT findings alone, and 11 in which the scan ruled out mediastinal proximity, preventing the need of additional testing. In a previous retrospective series from our institution [5], 14 stable patients with a transmediastinal GSW underwent a CT scan. Of these, 11 (78.5%) were successfully managed nonoperatively and 6 (42.8%) did not receive any further study.

The present series focuses on stable patients with a thoracic gunshot wound, rather than on patients with a proven mediastinal trajectory. This might explain the higher rate of patients with a successful nonoperative management of chest injuries, up to 90.3%. On the other hand, only a few number of additional imaging studies was necessary to evaluate mediastinal structures, since CT scan was able to exclude or diagnose significant injuries in the majority of cases. Indeed, in our experience the results of the initial CT scan were highly accurate in terms of identifying injuries requiring immediate operative interventions, and in all such cases an injury was identified and repaired operatively. Although the practice of confirming a suspicious vascular finding with a formal angiography has been abandoned at our center, all the major vascular injuries in this series diagnosed with CT scan were confirmed by surgical exploration. Moreover, and in contrast to the previous studies evaluating the accuracy of CT scan, in this series the presence of an isolated mediastinal hematoma, pneumomediastinum, or proximity injuries did not routinely prompt further diagnostic studies or surgery. In our experience the initial CT scan alone was able to exclude clinically significant injuries in most cases based on the direct imaging findings of a mediastinal injury or interpreted as highly suspicious for injury. The only significant injury missed on the initial CT scan was in the patient with multiple gunshot wounds involving both chest and abdomen who was transferred to the operating room immediately after imaging for an exploratory laparotomy due to severe intrabdominal injuries. Although the CT scan detected the mediastinal trajectory of the bullet, the involvement of the IVC, injured in its short intrathoracic portion, was not identified. The lesion was found intraoperatively and repaired primarily through a thoracotomy and the patient recovered to discharge. This is the only case of a falsely negative finding in our cohort of stable patients and does highlight that no imaging study should be considered infallible, and in particular the contrast timing to focus on the arterial phase may miss some major venous injuries.

In this review of the role of imaging in the decision-making process of penetrating thoracic patients, modern chest CT scan achieved an overall accuracy in the detection of injuries to mediastinal structures as high as 98.6%. In particular, the high negative predictive values obtained represent a reassuring finding in the scenario of a nonoperative management in patients with negative CT scan.

With regard to patients with indication to immediate surgical exploration after CT scan, median time from admission to surgery was 140 min. We believe that this represents a reasonable time, considering that all these patients were considered to be hemodynamically stable to undergo a radiologic evaluation after primary survey in the ED, while all other unstable patients underwent immediate surgical procedures and were excluded from this series. Moreover, the advantage of an accurate imaging evaluation whenever possible likely outweighs the risks of a relative delay in surgical exploration, as proven by the high accuracy that CT scan presented in indicating or excluding the need for surgery, without any increase in morbidity or mortality for patients requiring operation after CT scan that might be attributed to a delay due to the performance of diagnostic studies.

In the present study, none of the patients who underwent a delayed surgery after being selected for NOM were transferred to the operating room due to an injury missed on CT scan. Rather, the indications for delayed surgery were associated issues including retained hemothorax in patients with nonoperative management of thoracic bleeding, persistently high chest tube output in patients initially selected for nonoperative management, removal of foreign bodies found on CT scan, and clinical suspect of injuries for which CT scan has a low sensitivity. These included issues like bronchopleural fistula involving a peripheral bronchus and a diaphragmatic injury, that were thus further investigated with a minimally invasive thoracoscopic or laparoscopic exploration. Only a limited number of patients underwent further diagnostic workup, all negative, and angiography was always performed with a therapeutic intent during interventional radiology procedures. Overall, CT scan was used as standalone diagnostic tool in the majority of patients.

This study has several limitations, mostly resulting from its retrospective design. First, CT scan was not performed with a standardized protocol with regards to the timing of contrast injection, including both CT scan with arterial contrast phases and CT angiography to specifically enhance thoracic vasculature. Secondarily, in the absence of a specific diagnostic protocol, some studies to evaluate the esophagus and tracheobronchial tree were performed in patients without any suspicious finding on CT scan. Moreover, no injury to the aerodigestive tract was found in our cohort of stable patients, making impossible to determine the sensitivity of imaging in the diagnosis. Finally, since we considered all bullets reaching the chest as a potential threaten to mediastinal structures, all patients with a thoracic involvement were included, while the number of patients with a mediastinal involvement was relatively small.

## Conclusion

In our series, modern high-quality chest computed tomography provided highly accurate and reliable screening for penetrating thoracic trauma and those with potential or proven transmediastinal trajectory. We propose that CT scan may be effective as a standalone study in the majority of these patients or to guide further diagnostic workup, although a larger multicenter series would be required to definitively confirm this. Chest CT scan was also highly reliable as a disposition tool from the trauma bay and allowed for safe and successful nonoperative management in the majority of the cohort. Further prospective studies are needed to confirm the role of CT scan in the decision-making process in penetrating thoracic trauma, and particularly to focus on the subgroup of those with transmediastinal trajectory.

## Data Availability

De-identified data that support the findings of this study are available from the corresponding author upon reasonable request.
